# Design and methods for evaluating an early childhood obesity prevention program in the childcare center setting

**DOI:** 10.1186/1471-2458-13-78

**Published:** 2013-01-28

**Authors:** Ruby Natale, Stephanie Hapeman Scott, Sarah E Messiah, Maria Mesa Schrack, Susan B Uhlhorn, Alan Delamater

**Affiliations:** 1 Division of Education, University of Miami Miller School of Medicine, Miami, USA; 2 Division of Pediatric Clinical Research, University of Miami Miller School of Medicine, Miami, USA; 3 Division of Psychology, Department of Pediatrics, University of Miami Miller School of Medicine, Miami, USA; 4 Department of Epidemiology and Public Health, University of Miami Miller School of Medicine, Miami, USA; 5 Assistant Professor of Clinical Pediatrics, Department of Pediatrics, Division of Psychology, University of Miami Miller School of Medicine, Mailman Center for Child Development, Room #4010, Miami, FL, 33131, USA

**Keywords:** Childhood obesity, Methodology, School-based obesity prevention, Childcare centers, Young children

## Abstract

**Background:**

Many unhealthy dietary and physical activity habits that foster the development of obesity are established by the age of five. Presently, approximately 70 percent of children in the United States are currently enrolled in early childcare facilities, making this an ideal setting to implement and evaluate childhood obesity prevention efforts. We describe here the methods for conducting an obesity prevention randomized trial in the child care setting.

**Methods/design:**

A randomized, controlled obesity prevention trial is currently being conducted over a three year period (2010-present). The sample consists of 28 low-income, ethnically diverse child care centers with 1105 children (sample is 60% Hispanic, 15% Haitian, 12% Black, 2% non-Hispanic White and 71% of caregivers were born outside of the US). The purpose is to test the efficacy of a parent and teacher role-modeling intervention on children’s nutrition and physical activity behaviors. . The Healthy Caregivers-Healthy Children (HC2) intervention arm schools received a combination of (1) implementing a daily curricula for teachers/parents (the nutritional gatekeepers); (2) implementing a daily curricula for children; (3) technical assistance with meal and snack menu modifications such as including more fresh and less canned produce; and (4) creation of a center policy for dietary requirements for meals and snacks, physical activity and screen time. Control arm schools received an attention control safety curriculum. Major outcome measures include pre-post changes in child body mass index percentile and z score, fruit and vegetable and other nutritious food intake, amount of physical activity, and parental nutrition and physical activity knowledge, attitudes, and beliefs, defined by intentions and behaviors. All measures were administered at the beginning and end of the school year for year one and year two of the study for a total of 4 longitudinal time points for assessment.

**Discussion:**

Although few attempts have been made to prevent obesity during the first years of life, this period may represent the best opportunity for obesity prevention. Findings from this investigation will inform both the fields of childhood obesity prevention and early childhood research about the effects of an obesity prevention program housed in the childcare setting.

**Trial registration:**

Trial registration number: NCT01722032

## Background

One in four US children under age five is either overweight (≥85^th^ to <95^th^ percentile for age- and sex-adjusted percentiles for body mass index [BMI]) or obese (≥95^th^ percentile age- and sex-adjusted percentiles for BMI), with ethnic minority groups being disproportionately affected [1,2]. These statistics are of particular concern because obese preschool-aged children are five times more likely to be overweight during adolescence [3] and more than four times as likely to be obese adults when compared to their normal weight counterparts [3,4]. These results show that contrary to popular belief, children do not “grow out of” their “baby fat.” In fact, evidence indicates that excessive weight gain in the first years of life can alter developing neural, metabolic and behavioral systems in ways that increase the risk for obesity and chronic disease later in life [5,6]. Specifically, childhood obesity is a precursor to type-2 diabetes, cardiovascular disease, hypertension, stroke, osteoarthritis, asthma, and certain cancers [5,6]. As such, many reports have projected that childhood-onset obesity will contribute significantly to increased morbidity and mortality in adulthood [7], particularly among ethnic minority groups who are disproportionately affected by many of these chronic conditions [8].

Although few attempts have been made to prevent obesity during the first years of life, this period may represent *the* best opportunity for obesity prevention. During infancy and early childhood lifestyle behaviors that promote obesity are just being learned, and it is easier to establish new behaviors than to change existing ones. Furthermore, childcare settings offer a potentially very powerful opportunity to implement such efforts because; (1) 70% of preschool-aged children in our nation are enrolled in daily, out-of-home child care [9]; (2) children from low-income backgrounds consume 50%-100% of their Recommended Dietary Allowances (according to standards established by the Child Care Food Program) in the childcare setting; (3) many children spend the majority of their waking hours out-of-home, and in the childcare setting [10].

The majority of the childcare setting studies conducted to date focus on individual-level interventions [11-14] with few efforts targeting systemic policy-level changes that include evidence-based curricula and modifications to institutionally-provided meals and snacks. In fact, health and nutrition are often completely overlooked in the childcare setting because administrators and teachers feel they are under-qualified to deliver this information [15]. Additionally, many adults make food choices (purchasing and consumption) couched in personal attitudes and beliefs about food and nutrition, which in turn influence children’s nutritional beliefs, attitudes and behaviors [16,17]. The U.S. Department of Agriculture’s (USDA) concept of the “nutritional gatekeeper” and the “Project M. O. M. : Mother’s & Others & My Pyramid” campaign [18] suggests that empowering the nutritional gatekeepers in both the home and the child care center will potentiate a lasting and effective impact on the health and nutrition of future adults. For example, using a similar model in the elementary school setting “nutrition education extenders” were found to have a positive effect on child nutrition practices during and after the Healthier Options for Public Schoolchildren (HOPS) Study [19,20].

We describe here the research design and methods for a federally-funded, large-scale comprehensive obesity prevention intervention randomized controlled trial “Healthy Caregivers, Healthy Children HC2” to test (1) the utility of teachers and parents as effective nutritional gatekeeper role models, or change agents; and (2) the efficacy of center-based nutrition and physical activity policies, and how both affect early childhood growth patterns (namely body mass index), nutrition and physical activity practices among preschool-age children.

## Methods

A randomized, controlled obesity prevention trial in 28 low-income, ethnically diverse child care centers located throughout Miami-Dade County, FL is currently being conducted over two years (2010-present) to test the efficacy of an intervention that poises teachers and parents as lifestyle change agents. Trial methodology, intervention content and outcome measures are described in detail below.

### Study design

Because it was not feasible to randomize individuals within classrooms to different treatments, we randomly assigned centers to treatment or control conditions. Thus, HC2 is a group-randomized study, often referred to as a *cluster* randomized trial (CRT). In CRTs, clusters of people, or intact social units (such as childcare centers) rather than individuals are randomized to intervention and control groups and outcomes are measured on individuals within those clusters. CRT designs are used not only to evaluate group interventions but also individual interventions where group level effects are relevant. The University of Miami Institutional Review Board approved all aspects of the study.

### Inclusion criteria

Centers must have met the following criteria to be included in the study: (1) > 30 children ages 2-to-5 currently enrolled; (2) Serve low income families who are a part of the USDA food program and SNAP eligible; (3) Reflect the ethnic distribution of the Miami-Dade County Public School System (63 percent Hispanic, 19 percent African American, 18 percent white); and (4) Center director agrees to participate and sign a letter of commitment. Centers were excluded if they did not meet these criteria.

### Study participants

Parents consented to have their child participate in the study. A total of 60% of children were Hispanic, with the most prevalent specific Hispanic ethnicity being ‘Other’ (31%), followed by Cuban (15%), and Mexican (10%). Other specific ethnicities represented were Haitian (15%), non-Hispanic Black (12%) and non-Hispanic White (2%). Table 1 shows demographic characteristics of each study arm. There were 1105 participating children with 726 in the intervention arm and 379 in the control arm.

**Table 1:** Description of children participating in a childhood obesity prevention intervention trial by randomization arm

**Variable**	**Intervention (n=726, 66%)**	**Control (n=379, 34%)**
**Age, mean (SD) years**	4.21 (0.63)	3.74 (0.92)
**2-3 years, n (%)**	12 (2)	102 (27)
**3-4 years, n (%)**	275 (38)	104 (27)
**4-5 years, n (%)**	347 (48)	137 (36)
**More than 5 years**	92 (12)	36 (10)
**Race, n (%)**		
**White**	358 (56)	149 (49)
**African American**	198 (31)	119 (39)
**Other**	87 (13)	35 (12)
**Ethnicity, n (%)**		
**Hispanic Cuban**	71 (11)	70 (24)
**Hispanic Mexican**	83 (13)	7 (2)
**Hispanic Other**	235 (36)	92 (31)
**Haitian**	109 (17)	29 (10)
**Non-Hispanic Black**	57 (9)	60 (20)
**Non-Hispanic White**	13 (2)	11 (4)
**Other**	78 (12)	25 (9)

The majority of caregivers were born outside of the US (71%). Languages other than English were preferred by 65% of the sample. Low levels of education were prevalent, with 37% completing less than a 12^th^ grade education, 25% completed high school/GED, and 38% completing education beyond high school (Table 2).

**Table 2:** Description of caregivers of preschool children participating in a childhood obesity prevention intervention trial

**Variable**	**Total (n=980)**
**Age years (n=915), n (%)**	
18-24	137 (15)
25-30	339 (37)
31-40	350 (79)
41-50	79 (9)
+51	10 (1)
**Place of Birth (n=972), n (%)**	
USA	283 (29)
South America	93 (10)
Central America and Mexico	295 (30)
Caribbean Hispanic	140 (14)
Other Caribbean Islanders	154 (16)
Other	7 (1)
Years Lived in USA*, mean (SD)	11.96 (7)
**Language/s Spoken at home n (=978), n (%)**	
Only English	198 (20)
Only Spanish	354 (36)
Only Creole	32 (3.5)
English plus other language	393 (40)
Other	4 (0.50)
**Preferred Language (n=975), n (%)**	
English	369 (38)
Spanish	428 (44)
Creole	76 (8)
French	6 (1)
Other	96 (9)
**Education (n=949), n (%)**	
Didn’t complete high school	348 (37)
Completed high school or GED	238 (25)
Any education higher than high school	363 (38)

## Study procedures

### Intervention arm

Treatment or intervention arm schools received a combination of (1) implementing a daily curricula for teachers/parents (the nutritional gatekeepers); (2) implementing a daily curricula for children; (3) technical assistance with meal and snack menu modifications such as including more fresh and less canned produce, no high fat milk, and less simple carbohydrate items; and (4) creation of a center policy for dietary requirements for meals and snacks, physical activity and screen time. Details of each of these components follows.

### Teacher/parent nutritional gatekeeper curriculum

For childhood obesity prevention programs to achieve long-term success the nutritional gatekeeper (i.e. the person buying and supplying food for the children) must engage in healthy lifestyle behaviors and beliefs themselves. For young children the nutritional gatekeeper is most likely the parent or a primary caregiver and for those who attend childcare, the teachers and center staff also. Therefore, Curriculum Specialist (CS) utilized an integrated curriculum with teachers and parents at the child care centers. The teacher-focused curriculum consisted of 29 weekly technical assistance sessions delivered by HC2 curriculum specialists during weekly visits to the childcare centers. Additionally, HC2 program staff conducted joint parent-teacher meetings (providing a substitute so that teachers can attend during parent pickup times) that focused on an evidence-based nutrition and physical activity curriculum. CS taught parents and teachers about (1) Choosing healthy foods, with an emphasis on fruits and vegetables; (2) Making healthy food choices for infant and child development; (3) Preparing nutritious snacks; (4) Preparing new recipes; (5) Tasting new foods; (6) Learning about food safety and storage; (7) Planning meals; (8) Making grocery lists; (9) Shopping wisely for groceries; (10) Budgeting food dollars and food stamps; and, (11) Using food labels to choose best buys. Via role-modeling, parents and teachers were encouraged to implement change at the family/home and child care center level, not child level. Additionally, nutritional professionals served as role models for the teachers and parents and assisted them as nutritional gatekeepers and positive models for the children. Lessons on physical activity for the whole family were also included (Table 3).

**Table 3:** Nutritional gatekeeper role modeling curriculum lessons, objectives and activities for childcare center teachers and parents

**Lesson**	**Objectives, To Learn:**	**Activities**
**Lesson 1:** If you are active, your child will be active.	· the benefits of being an active caregiver and child	Caregivers fill out the “Together We Can” weekly Caregiver and child activity chart
	· the right conditions for your child to have the best time while exercising with you	Find local and inexpensive physical activities in the area that families can do together
	· the “Three Elements of Physical Fitness” in action in the playground	
	· physical activities that strive for endurance, strength, and flexibility that can be done with your child	Perform endurance, strength, and flexibility exercises with caregivers
	· how to be a good example for your child	
	· why physical activity is important for you and your child	
	· how much physical fitness your child needs	
**Lesson 2:** Empowerment and problem solving : Staying active and having a positive body image	· Fun exercises you can do with your child	Caregivers will take the “Healthy Body Survey” and discuss the results
	· What body image is and how you can develop a better one	
	· How to become a healthy eater	
	· How to determine if you have a healthy relationship with food and fitness	Discuss and perform activities that keep caregivers and children active at home
**Lesson 3:** If you eat healthy foods, your child will eat healthy foods	· How to get kids to chose what they like to eat	Prepare a kid-friendly fruit and veggie party and taste the goodies
	· The benefits of taking kids to the grocery store	
	· The benefits of cooking with your kids	Demonstrate how children can take part in cooking a healthy, easy and quick meal at home.
	· How to get kids to try new foods	
	· Tips on fighting obesity	
	· Fun ideas for healthy eaters	
	· How much to feed your child	
	· Eating safety tips	Show healthy portion sizes for preschoolers
**Lesson 4:** Empowerment and Problem Solving: Staying active	Tips on how to have a fit child	Perform yoga exercises for caregivers and children
	· Tips on fighting obesity and staying active	
	· How to help children learn new skills while going for a walk	Play fun, active games that can be played at home
	· What preschool children should be able to do	
	· When you should call a doctor	

Our framework was comprised of a variety of role modeling components from the USDA’s Project M.O.M [18,21-24]. These components were then adapted and merged into eight sessions that were appropriate for the parents/caregivers and teachers of pre-school aged children. All curriculum materials if not available in Spanish were translated and back translated into Spanish by a team of certified translators at the University of Miami.

Teachers received ongoing technical assistance to support the inclusion of healthy eating and physical fitness concepts in their weekly lessons, without the continued assistance of the CS, and so, during the second year of the study they could lead the lessons independently or without any CS assistance. Currently, in year two, booster trainings are being provided bimonthly in all centers to provide teachers with refresher overviews of all lesson plans.

Parents participated in monthly events such as dinners at the centers where they received the same nutrition and physical activity educational information that formed the content of the teacher curriculum. They also received monthly newsletters that contained information that was covered in that month’s school lessons and an overview of the center’s HC2 activities for that month.

### Child curriculum

Twenty lesson plans were developed to promote nutrition and physical activity and were supplemented with the OrganWise Guys (OWG). The lesson plans focused on core principles of healthy living (high fiber, low fat, lots of water, exercise) and eating (nutrient-dense foods), nutritious dietary offerings in USDA meals that model classroom-based and parent nutrition education programming via the USDA feeding and nutrition education programs and has been shown to have positive health outcomes among primary school children [19,20].

This classroom-based curriculum consists of 20 weekly sessions that were provided to the teachers by project curriculum specialists (CS) during weekly visits. The teachers then taught the curriculum to the children. The CS would address menu changes and target the cognitive, cultural, and environmental barriers to a low fat, high fiber diet that include more fruits and vegetables. These weekly visits are designed to assist center staff in problem-solving ways to ensure implementation. In addition, the curriculum was translated and subsequently implemented in Spanish where applicable.

### Technical assistance and creation of center policies

All center modifications were revenue neutral (do not have an additional cost). The meals, snacks and beverages were provided by caterers who were given technical assistance by the HC2 nutritionist to ensure that the meals followed the policy guidelines yet still met USDA nutrition requirements. Center administrators were given monthly technical assistance by both the project nutritionist and staff on menu development and curriculum enhancements that would include the integration of the nutritional gatekeeper curriculum [3,25-27] critical for intervention maintenance and effective mediators of change.

### Beverage policy

Menus in intervention centers were modified to include low-fat (1%) or skim milk, water, less juice. Low fat or skim milk was promoted only at lunch, and water was promoted as the primary beverage for staff and children, especially after physical activity.

### Snack policy

Menus in intervention centers were modified to include more fresh fruit (or canned fruit in water or 100% juice), fresh vegetables (or low-sodium canned vegetables), and fewer simple carbohydrate snacks (cookies, crackers, etc.). The centers also were encouraged to incorporate fresh fruits and vegetables for snack and meal time as often as possible. In addition, we provided food tastings to the children to expose them to new fruits and vegetables that they may have never tried before (e.g. kiwi, blackberries, rhubarb, edamame, and cauliflower).

### Physical activity policy

Physical activity was promoted for at least 60 minutes per day. Lesson plans offered structured physical activities in the classrooms and included music and movement CDs with songs and exercises. Teachers were also provided with rainy day activities that could be used in the classroom and equipment that can be purchased inexpensively at local stores (jump ropes, hula-hoops, balls, etc.) but were encouraged to conduct physical activities outside and incorporate free play as much as possible.

### Reduction of screen time policy

 The reduction of screen time was addressed by limiting television viewing, watching movies, and playing computer games to 30 minutes or less each day. The amount of time screens was documented.

### Control arm

Those schools randomized to the control arm received a safety curriculum and some child care center locations received an attention control consisting of one visit from the Injury Free Mobile which provided parents and teachers with home, car and child seat safety information. The control group received all the same pre-post measures (see below) as the intervention arms. They also received the same incentives as the intervention arms to foster involvement and ensure retention/reduce loss to follow up.

## Measurements

All measures were administered at the beginning and end of the school year for year one and year two of the study for a total of 4 longitudinal time points for assessment. All measures were included to measure pre-post intervention changes in nutrition and physical activity knowledge, attitudes, and beliefs, defined by intentions and behaviors. Anthropometric measures were also included. Effort was made to maximize assessment sensitivity to functional domains deemed most critical to obesity prevention research and with strong consideration for test validity and reliability, availability of recent multiethnic normative samples, and age ranges avoiding floor/ceiling effects. In addition, each of the instruments selected has either been (a) standardized on culturally, racially and social economically diverse populations or (b) used in research projects involving culturally and racially diverse populations. A summary of all measures are included in Table 4 and their procedures are described briefly below. 

**Table 4:** Primary and secondary study outcomes and accompanying measurement tool for each study variable and participant

**PRIMARY OUTCOMES: CHILD HEALTH MEASURES**	**DOMAINS**
**Anthropometric Variables.** Assessment of body composition includes height (by stadiometer), weight (by digital scale), body mass index (BMI, defined as weight in kg/height in meters squared). Participants are asked to remove their shoes and any heavy outer clothing prior to measurement to avoid error. A total of three measures are taken at each data collection point and then an average of these three are used for the analysis.	**Variables:** Height. Weight, BMI, waist circumference
	**Participant:** Child
	Time: 5 mins
**Healthy Kids Checklist**. This instrument is a 32-item rating scale that measures nutrition behaviors and physical activity habits at home of both parent and child. It also evaluates parental role modeling of healthy lifestyle practices. The Checklist is targeted at children in preschool in low-income areas served by the USDA food program. The parent serves as a reporter for a child’s lifestyle (physical activity, sleeping habits, etc.) and eating behaviors. This scale is comprised of three domains consisting of 12 determinants that should be included in a pediatric obesity risk assessment tool: 1) *Diet* domain includes eight determinants: fat, fiber, fruit/vegetable intake, calcium/dairy intake, sweetened beverages, eating in restaurants, breakfast skipping, energy density; 2) *Lifestyle* domain includes three determinants: physical activity, sleep duration, television viewing. 3) For the *Parenting* domain, role modeling is determined. Parent-report of child physical activity also includes TV viewing. In addition, it utilizes low-literacy, pictorially representing items and has been validated for this population of low income families.	**Variables:** Child and Parent Home Diet, Home Lifestyle, Parenting, Role Modeling
	**Respondent:** Parent proxy
	Time: 10 mins
**Willett Semiquantative Food Frequency Questionnaire.** To measure changes in food items served to children by the childcare centers, a food frequency questionnaire has been adapted from the Willett Semiquantative Food Frequency Questionnaire (1998). This will be completed by the owner of the facility about types of foods offered. The survey is available in English and Spanish and is a reliable and valid measure. Owners are asked to provide a copy of the invoice of food ordered that month along with the questionnaire.	**Variable:** Childcare Center Diet
	**Respondent:** Center Director proxy
	Time: 5 mins
**MATERNAL and CHILDCARE PROVIDER MEASURES**	**DOMAINS**
**Descriptive Information.** A comprehensive survey has been previously developed that includes demographic questions such as age of the child and respondent; perceptions regarding the weight of the child as well as the respondent; perceived health status; country of origin of the child and respondent; language spoken in the home; food consumption patterns; acculturation questions related to food purchases and preferences; and food insecurity will be implemented. This survey has been adapted using valid measures and is culturally appropriate for the Miami-Dade community based on ethnicity.	**Variables: Food insecurity,** Acculturation
	**Respondent:** Parent, Teacher
	Time: 15 mins
**Food Behavior Checklist (FBC).** A 16-item self-report measure of parent and teacher dietary intake, this tool includes seven fruit/vegetable, two milk/dairy, one food security, three diet quality and three fat/cholesterol items. With a sample of low-income clients from eight counties participating in EFNEP and FSNE, authors reported a criterion validity coefficient (r =.43) with a biomarker, serum carotenoids, for the fruit and vegetable sub-scale. Convergent validity was reported for 10 selected nutrients and the Healthy Eating Index for the 16-item tool. Compared with the 24-hour dietary recall, it is less time-consuming to administer and analyze, with a reduced respondent burden. Responses to nineteen food behaviour items were significantly correlated with hypothesized 24 h recall data (with a maximum correlation of 0·44 for drinking milk and calcium) or the USDA HFSSM (0·42 with the food security item). Coefficients for test–retest reliability ranged from 0·35 to 0·79. Cronbach’s *α* ranged from 0·49 for the diet quality sub-scale to 0·80 for the fruit and vegetable sub-scale.	**Variables**: Diet and Lifestyle (planning and fixing food)
	**Respondent:** Parent, Teacher
	Time: 10 mins
**Physical Activity Self-Efficacy Scale.** This five-item questionnaire–measures confidence regarding participation in physical activity such as overcoming barriers to exercise including negative affect, relapse, lack of time, and environmental conditions. The measure utilizes an 11-point scale ranging from “not at all confident” to “very confident”. Test-retest (product moment) reliability over a two-week period was .90. The instrument has also demonstrated adequate concurrent validity with stages-of-change measures.	**Variables:** Self-efficacy for being physically active
	**Respondent:** Parent, Teacher
	Time: 5 mins
**CLASSROOM ENVIRONMENT**	
**Lesson Plan Review of Physical Activity.** Classroom schedules and lesson plans will be reviewed to measure time spent in physical activity. This information will assist with determining fidelity to the treatment model. Data will be collected by trained RAs utilized the tool developed in our pilot study and include items such as amount of time spent in outdoor play, amount of time spent watching TV, etc.	**Variables:** Classroom physical activity environment
	**Respondent:** Observe
**Behavior Observation of Classroom.** Data will be collected by trained RAs on foods and drinks served in the classroom. This will consist of observing breakfast, lunch, and snack and will determine fidelity to the treatment intervention (i.e. Children are included in preparation of meals as much as possible, staff serve as healthy role models, canned fruits are low in sugar and drained before being served). We have successfully implemented this tool in both our HI HO and HC2 projects.	**Variables:** Classroom nutrition environment
	**Respondent:** Observation

## Child health measures

### Anthropometric outcomes

Height (by stadiometer), weight (by digital scale) and waist circumference measurements were collected at the beginning and the end of year one and two school year. Children’s shoes and any heavy outer clothing prior to measurement. BMI is converted to an age and sex- adjusted percentile and z-score [28]. According to the CDC, the following percentile categories will be used to define weight groups; underweight (< 5^th^ percentile), normal weight (≥ 5^th^ to < 85^th^ percentile), at risk for overweight (≥ 85^th^ to < 95^th^ percentile ) and overweight (≥ 95^th^ percentile) [29].

### Physical activity level

The *Healthy Kids Checklist*[30-32] was administered to measure nutrition behaviors and physical activity patterns at home of both parent and child. It also evaluates parental role modeling of healthy lifestyle practices. The parent serves as a reporter for a child’s lifestyle (physical activity, sleep duration, television viewing, and presence of a television in the child’s bedroom) and eating behaviors.

### Nutrition

Child nutritional behaviors are assessed with two measures. *Healthy Kids Checklist*[30-32], which includes 8 determinants: fat, fiber, fruit/vegetable intake, calcium/dairy intake, sweetened beverages, eating in restaurants, breakfast skipping, energy density. This measured (by parent proxy) child nutritional behaviors at home and the *Food Frequency Questionnaire* (FFQ) [33] measures nutritional behaviors at school. The FFQ has been adapted from the Willett Semiquantitative Food Frequency Questionnaire [34] to measure monthly changes in food items served to the children by the childcare centers. Additionally, center directors provided a copy of the invoice of food ordered for the month accompanying the questionnaire.

## Parent and childcare provider nutrition and physical activity measures

### Nutrition

The following two instruments assessed adult nutritional behaviors; (1) the *Food Behavior Checklist*[30] is a short, culturally neutral, valid and reliable indicator of fruit and vegetable consumption. The 16-item checklist includes 7 fruit/vegetable, 2 milk/dairy, 1 food security, 3 diet quality and 3 fat/cholesterol items. Convergent validity was reported for 10 selected nutrients and the Healthy Eating Index for the 16-item tool [30,31]; and (2) the *Fruit and Vegetable Inventory*[32], an instrument that contains 13 psychosocial items shown to be related to fruit and vegetable intake. The six constructs are perceived control, self-efficacy for eating healthier, readiness to eat more fruit, readiness to eat more vegetables, and perceived diet quality.

Adult health literacy is measured using the *Pediatric Health Literacy Activities Test*[35] a 10-item instrument validated in English and Spanish among caregivers of young children. It correlates well with lengthier measures of literacy and numeracy. It includes items related to growth and nutrition (e.g., infant formula mixing, nutrition labels), as well as general health items.

### Physical activity

A modified (only the 4 items relating to physical activity were administered) version of the *CATCH Physical Activity SelfEfficacy Questionnaire*[36] measures parent’s self-efficacy to be physically active.

### Maternal/Caregiver characteristics

Baseline characteristics for children, parents and childcare providers were collected and included infant feeding history (breastfeeding exclusivity and duration, onset of solid foods, age of weaning from bottle), and parent perception of child weight status, acculturation level, and level of food insecurity in the household.

### Classroom environment

Time spent in daily physical activity, classroom schedules and lesson plans, and foods and drinks (items, amounts) and monthly behavior observations are recorded by study staff for each classroom.

## Process measures

### Parent activity survey

To track the amount of dinners parents attend, activities parents complete, and number of newsletters that are received/read, parents are given an annual survey that includes a satisfaction component inviting parents to give feedback and to rate the helpfulness of each activity.

### Teacher process survey

Process data is collected to determine the feasibility of implementing HC2 on a larger scale and to determine the sustainability of the project. The CS complete a weekly process worksheet for each center by interviewing teachers. This information will help answer the larger feasibility questions of: 1) What are the range of common and uncommon barriers to implementing an intervention like HC2? 2) What is the range of creative solutions to overcoming these barriers? 3) What are the unique assets of child-care centers that enable them to adapt such an intervention? 4) What key community assets may be necessary to bring an intervention like HC2 “to scale” across the entire county?

## Power analysis

Our power analysis indicates that with 80% power (alpha = 0.05, two tailed) we will need 12 centers per group/cluster (2 groups/clusters) for a total of 24 centers and 30 individuals per center for a 1.10 effect size to give us confidence in our statistical analysis that findings are not due to chance alone.

## Statistical analysis

### Univariate/Bivariate

Our univariate analysis consists of simple descriptive measures such as percentages, medians, ranges, variances, and standard deviations to describe all study participants and sites. Bivariate analysis is used to explore possible confounding variables such as age, gender and ethnicity via simple contingency table analysis and ANOVA. Pearson product moment correlation coefficient matrixes investigate the relationships (either positive or negative) in the percent change in BMI and all predictor variables. We pool data within-cells to account for our randomized group study design that essentially blocks at the center level (PROC DISCRIM procedure in the SAS statistical package). ***Multivariate*.** Repeated measures linear regression models are used to determine patterns of progression or improvement in BMI percentile over time, as well as to identify demographic and family characteristics associated with BMI outcomes. Repeated measures analysis of covariance (ANCOVA) determines the relationships between BMI and our environmental and family modification (via the theoretical constructs) covariates. ANCOVA is used to increase power in a one-way or two-way ANOVA by adding a second or third variable as a covariate. ANCOVA was an attractive higher level method of analysis for our design because it is capable of removing the obscuring effects of pre-existing individual differences among subjects while also allowing for compensation for systematic biases among our samples. **Cost Analysis**. Resource use and cost data for the intervention are being collected across all intervention centers. Key statistics from the cost evaluation will include total annual economic cost for the intervention and weekly economic cost per child.

## Discussion

*Healthy People 2020*[37] identifies “Nutrition and Weight Status” as one of their major objectives to “promote health and reduce chronic disease risk through the consumption of healthful diets and achievement and maintenance of healthy body weights.” This objective emphasizes that efforts to change diet and weight should address the policies and environments that support these behaviors in settings such as schools [37]. Although few attempts have been made to prevent obesity during the first years of life, this period may represent the best opportunity for obesity prevention. Findings from this investigation should inform both the fields of childhood obesity prevention and early childhood research about the effects of an obesity prevention program housed in the childcare setting. As childhood obesity continues to be a topic of national interest is in the best interest of health care providers, educators, and parents to begin thinking about this as a priority for ensuring the healthy future of our community’s children.

The Institute of Medicine’s (IOM’s) Standing Committee on Childhood Obesity Prevention recently commenced a study to examine the evidence and provide guidance on obesity prevention policies for children ages birth to five years old. Based on this study’s findings, in 2011 this committee created a set of policy recommendations designed specifically to prevent obesity in early childhood by promoting healthy early environments in settings outside the home where young children spend substantial time, namely childcare settings in particular [38]. Recommendations were formulated using the best evidence available, including both direct (published literature, organizational reports) and indirect (expert input) data about the likely impact of a given policy on reducing childhood obesity. The committee’s overreaching goal was to have the report “find its way to federal, state and local government policy makers who work in areas that impact young children.” However, they acknowledge that “it will be important to act today based on what is known, while also undertaking the necessary research and policy evaluation to ensure better informed and effective actions in the future.” To date, the concept that incorporating these policies in the childcare setting will result in reduced obesity rates, increased access to nutritious foods, increased physical activity and decreased inactivity has been largely based on conjecture, not on scientific evidence produced by adequately controlled trials, making this study all the more timely.

## Limitations and strengths of study design

We are limited in our ability to generalize our research findings to children who are older than 5 years and to families that are not from low income backgrounds. Strengths of the study include the design, as randomized controlled trials are considered the “gold standard” in terms of design rigor and ethnic diversity of the sample.

## Conclusions

Although few attempts have been made to prevent obesity during the first years of life, this period may represent the best opportunity for obesity prevention. Findings from this investigation should inform both the fields of childhood obesity prevention and early childhood research about the effects of an obesity prevention program housed in the childcare setting.

## Abbreviations

BMI: Body mass index; CS: Curriculum specialists; HC2: Healthy Caregivers Healthy Children; OWG: Organ Wise Guys; USDA: United States Department of Agriculture.

## Competing interests

No conflict of interest to report for all authors.

## Authors’ contributions

RN conceived of the study, and participated in its design and coordination and helped to draft the manuscript. SHS assisted with data collection and helped draft the manuscript. SM participated in the design of the study, performed the power analyses, and helped draft the manuscript. MS carried out the data collection. SU assisted with Internal Review Board approval and data collection procedures. AD participated in the design of the study. All authors read and approved the final manuscript.

## Pre-publication history

The pre-publication history for this paper can be accessed here:

http://www.biomedcentral.com/1471-2458/13/78/prepub
